# Rescuing the Host Immune System by Targeting the Immune Evasion Complex ORF8-IRF3 in SARS-CoV-2 Infection with Natural Products Using Molecular Modeling Approaches

**DOI:** 10.3390/ijerph19010112

**Published:** 2021-12-23

**Authors:** Aqel Albutti

**Affiliations:** Department of Medical Biotechnology, College of Applied Medical Sciences, Qassim University, Buraydah 52571, Saudi Arabia; as.albutti@qu.edu.sa

**Keywords:** ORF8, IRF3, immune evasion, virtual screening, molecular dynamics simulations

## Abstract

The perennial emergence of SARS-CoV-2 and its new variants causing upper respiratory complexities since December 2019 has aggravated the pandemic situation around the world. SARS-CoV-2 encodes several proteins among which ORF8 is a novel factor that is unique to SARS-CoV-2 only and is reported to help the virus in disease severity and immune evasion. ORF8-IRF3 complex induces endoplasmic reticulum stress, thus helps in the evasion of immune response. Consequently, targeting the ORF8-IRF3 complex is considered as a prime target for the discovery of novel drugs against SARS-CoV-2. In this regard, computational methods are of great interest to fast track the identification and development of novel drugs. Virtual screening of South African Natural Compounds Database (SANCDB), followed by docking and molecular dynamics (MD) simulation analysis, were performed to determine novel natural compounds. Computational molecular search and rescoring of the SANCDB database followed by induced-fit docking (IFD) protocol identified Quercetin 3-O-(6″-galloyl)-beta-D-galactopyranoside (SANC00850), Tribuloside (SANC01050), and Rutin (SANC00867) are the best scoring compounds. Structural-dynamic properties assessment revealed that these three compounds have stable dynamics, compactness, and a higher number of hydrogen bonds. For validation, we used MM/GBSA, in silico bioactivity estimation and dissociation constant (K_D_) approaches, which revealed that these compounds are the more potent inhibitors of the ORF8-IRF3 complex and would rescue the host immune system potentially. These compounds need further in vitro and in vivo validations to be used as therapeutics against SARS-CoV-2 to rescue the host immune system during COVID-19 infection.

## 1. Introduction

The perennial emergence of SARS-CoV-2 and its new variants causing upper respiratory complexities since December 2019 has aggravated the pandemic situation. This pandemic has caused 238,378,962 mortalities and 4,863,187 deaths worldwide as of 10 October 2021. This virus causing coronavirus Disease-19 (COVID-19) with a single strand RNA is continuously subjected to evolution by bringing new mutations [1,2]. The genomic organization of this virus shares commonalities with other beta coronaviruses. The six functional open reading frames (ORFs) are well-arranged in order from 5′ to 3′ replicas (ORF1a/ORF1b), membrane (M), envelope (E), spike (S), and nucleocapsid (N). In contrast, the nonstructural proteins include 3-chymotrypsin such as protease, papain-like protease, and RNA-dependent RNA polymerase, which are encoded by the ORF regions [3,4,5]. Among these, spike protein is essential which mediate cellular attachment and entry to the cell and starts the infections. ORF8 is a novel factor that is unique to SARS-CoV-2 only and is reported to help the virus in disease severity and immune evasion [6,7].

ORF8 with 121 amino acids display captivating features and is more susceptible to mutations. Among the key events regulated by ORF8 involve downregulation of type I interferon signalling and histocompatibility complex class I (MHC-I) pathways [8,9]. Many studies reported the interaction of ORF8 with IRF3 to hijack the host immune system and potentially evade [10]. Interaction of ORF8-IRF3 is reported to target different aspects of the IFN signaling cascade, thus suppressing the host immune system and proceed a successful infection. Many studies also reported mutations in ORF8 such as W45L, V62L, and L84S structurally characterized are reported to more robustly evade the host immune system [6]. ORF8 is also reported to form intracellular aggregates in the Epithelial Cells of the lungs. It is also reported to induce endoplasmic reticulum stress, thus helps in the evasion of immune response [7]. Thus, targeting the ORF8-IRF3 is considered as a prime target for the discovery of novel drugs against SARS-CoV-2 [11].

Due to the multi-faceted role of ORF8-IRF3 complex, it is important to abolish this complex either with small natural molecules, peptides inhibitors, or nanobodies. In this regard, computational methods are of great interest to fast track the identification and development of novel drugs [12,13,14,15,16,17,18]. Using computational methods various drugs for other targets, vaccines, peptides, and nanobodies are discovered against SARS-CoV-2 [19,20,21,22,23]. Thus, employing the same approach here would greatly assist the drug development cycle against this immune evasion complex.

Herein we used computational modeling and simulation approaches to target the interface of the ORF8-IRF3 complex with high-affinity natural products [24]. South African Natural Compounds Database (SANCDB) (http://sancdb.rubi.ru.ac.za/ (accessed on 10 October 2021) [25] containing compounds from plant sources and marine life was screened against the interface residues of IRF3. Computational molecular search and rescoring of the whole database followed by induced-fit docking (IFD) protocol were employed to identify the best scoring compounds. All-atoms simulation and binding free energy approaches were then applied to validate the potential of these compounds. Finally in silico bioactivity prediction and dissociation constant (K_D_) approaches validated the efficacy of these final hits. In sum the shortlisted compounds here provide basis for the development of next-generation drugs focusing on the host factors to rescue the host immune system.

## 2. Materials and Methods

### 2.1. IRF3, Database Retrieval and Preparation

The crystal structure of IRF3 available online was retrieved from RCSB using accession ID 1QWT. The retrieved structure was prepared, refined, and minimized using Chimera and GalaxyRefine (USCF, CA, USA) webserver [26,27,28].

### 2.2. Drugability Assessment of the Binding Interface

To find the druggable pockets in the binding interface of IRF3, the DoGSiteScorer web server is freely available at http://dogsite.zbh.uni-hamburg.de (accessed on 10 October 2021) was used [29]. The servers predicts the druggability potential of the interacting interface of the two proteins.

### 2.3. Molecular Search against ORF8-IRF3 Interface

Identification of potential natural compounds through a molecular search for IRF3, using SANCDB database. All the drugs, 1200 total including flavonoids, alkaloids, phenylpropanoids, polyketides, and terpenoids in the database were prepared and minimized using PyRx tool [30]. The interface residues 189–240 of IRF3 required for interaction with ORF8 were selected as reported by a previous literature [6]. For instance the role of medicinal natural compounds such as flavonoids, alkaloids, phenylpropanoids, polyketides, and terpenoids is very obvious against SARS-CoV-2 and has been previously reported by various studies [12,13,20,31]. Screening against the binding site residues was performed, and the best scoring hits were selected for re-docking and rescoring.

### 2.4. Rescoring and Ranking Using Induced-Fit Docking (IFD) of the Top Hits

The best scoring compounds were re-evaluated using IFD protocol using 64 exhaustiveness to remove and false-positive results and confirm the final hits. AutoDockFR–AutoDock for Flexible Receptors (ADFR) [32] based on AutoDock4 scoring function was used to re-score the best hits using IFD algorithm. This approach minimizes the internal energy of the receptor and achieves the receptor sidechain best conformation by employing 14 different sidechains to boost the frequency of accurate docking. In comparison with AutoDock Vina, AutoDockFR attained higher accuracy in a cross-validation check, and also, the speed of the docking is much higher.

### 2.5. Validation of the Top Hits

#### 2.5.1. Molecular Dynamics Simulation of the Top Hits

Structural-dynamic features of the top hits were explored through all-atoms simulation using the AMBER18 package [33]. The Amber general force field (GAFF) and ff14SB forcefields were employed for drugs and IRF3 topologies. Solvation with TIP3P water box and CL- ions were added for neutralization of each system. Energy minimization of each complex was achieved in two steps, followed by heating and equilibration. For the annotation of long-range electrostatic interactions, a Particle Mesh Ewald (PME) algorithm [34] and 1.4 nm cut-off value for vdW and Columbic interactions of short-range was set. For temperature regulation, Langevin thermostat (300 K) and Berendsen barostate were used for pressure control. A time step of 2fs and a total simulation time of 200 ns for each complex were performed. The dynamic stability, flexibility, compactness to show the binding and unbinding events of the drugs in the cavity, and other features of the ligands-protein complexes were calculated by utilizing CPPTRAJ and PTRAJ modules [35].

#### 2.5.2. The Binding Free Energy Calculations

The top hits in complex with IRF3 were subjected to binding free energy calculation using the script MMPBSA.PY by the whole trajectory [36,37,38,39]. Various studies have employed this method to compute the TBE of different compounds [40,41,42,43,44].

ΔG*_bind_* = ΔG*_complex_* − [ΔG*_receptor_* + ΔG*_ligand_*]

Here, ΔG*_bind_* stands for the total free binding energy, while others denote the free energy of the protein, the ligand and complex. The equation given below was then used to estimate each specific energy term that contributes to the total free energy:

G = G*_bond_* + G*_ele_* + G*_vdW_* + G*_pol_* + G*_npol_*

Bonded, electrostatic, polar, non-polar, and van der Waal energy terms are represented by the above equation.

#### 2.5.3. In Silico Bioactivity Prediction

To validate the binding of the top hits, we predicted the bioactivity of each compound against different classes of proteins using the Molinspiration web tool. The scores between −0.5 to 0.5 demonstrate excellent inhibitory activity.

#### 2.5.4. Dissociation Constant (K_D_) Evaluation

Binding kinetics is an important parameter to estimate the strength of the association of drug and a protein and is widely used by a larger number of studies. Herein, to estimate the dissociation constant of the top hits, we used PROtein binDIng enerGY prediction (PRODIGY) server (https://wenmr.science.uu.nl/prodigy/ (accessed on 18 October 2021)) [45].

## 3. Results and Discussion

### 3.1. Structural Retrieval and Analysis of Druggable Site

**Figure 1:**
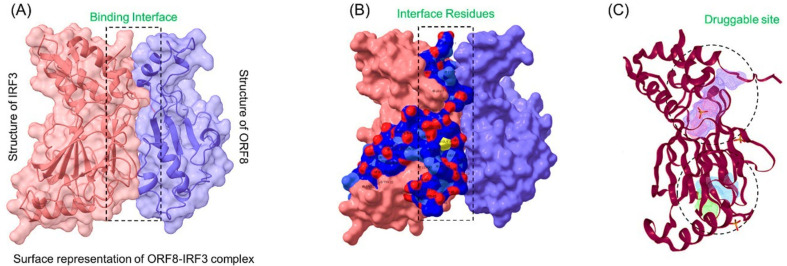
Representation of the ORF8-IRF3 complex. (**A**) Surface representation of the ORF8-IRF3 complex, (**B**) interface residues of IRF3 while (**C**) Druggable site on IRF3 protein.

The pandemic caused by SARS-CoV-2 has troubled the world by spreading exponentially to every nook and cranny of the world. The pathophysiology of this virus demonstrates that it principally affects the upper respiratory canal by entering the host cell through spike protein. The adherence of the spike protein, receptor-binding domain to the host ACE2 protein, allows the entry to the host cell, and it is considered the primary drug target for novel drug discovery [46,47]. However, the role of other proteins, particularly the ORF8-IRF3 complex, is very important in the disease’s severity and immune evasion. Therefore, abrogation of this complex would rescue the host immune system and thus will ensure the efficient neutralization of the virus. Hence, drugs that can target this complex are required to rescue the host immune system. The current study uses state-of-the-art methods to search potential drugs from medicinal plants sources using genetic algorithm, IFD-algorithm, all-atoms simulation, binding free energy calculation, bioactivity prediction, and dissociation constant determination. Herein, we targeted the IRF3 interface residues (189–240) using SANCDB database, which contains drugs from natural plants and marine resources. The complex of ORF8-IRF3 was obtained through a protein-protein docking approach to confirm the binding interface with a previous study [6]. Our analysis revealed the same binding interface for both the structures shown in Figure 1A. The interface residues of IRF3 were then subjected to druggability assessment which revealed that the binding interface is a druggable site and can be targeted using drugs. The druggable residues are shown in Figure 1B,C.

### 3.2. Ranking the Best Compounds and Rescoring

Keeping in view the importance of ORF8-IRF3 complex in immune evasion, we screened phytomedicines from natural plants and marine sources. Using a multi-steps screening approach, we ranked the best compounds which could abrogate the IRF3 interaction with ORF8. In the first round, the whole database was screened using PyRx. The results revealed the docking scores range from −8.23 to −4.11 kcal/mol. Then we screened the best compounds with docking score >−6.0 kcal/mol. With this criterion, only 202 compounds were shortlisted for rescoring and analysis. In the second step, AutoDock Vina-based docking of these 202 compounds was performed, and the docking score range from −8.52 to −4.83 kcal/mol. Finally, using ADFR, IFD docking of the top 10% compounds were redocked and scored. The docking score for these 41 compounds using IFD approach range from −10.52 to −4.97 kcal/mol. Among these compounds with good docking scores were selected, which shortlisted only six compounds to have the best scores and interactions. These compounds include SANC00524 (−10.52 kcal/mol), SANC00526 (−9.53 kcal/mol), SANC00850 (−6.93 kcal/mol), SANC01085 (−6.37 kcal/mol), SANC00867 (−6.27 kcal/mol) and SANC00395 (−5.98 kcal/mol). The top six hits were then checked for Lipinski’s rule, which revealed that only three compounds SANC00850, SANC01085, and SANC00867 passed the criteria and were subjected to further analysis while the rest were discarded. The docking scores, database ID, 2D structure, and names of these compounds with the highest affinity towards the protein are given in Table 1. The binding mode of each compound is given in details below. These three compounds can be seen that they all possess almost similar scaffold with benzene rings surrounded by hydroxyl groups which make it possible for many interactions with the residues.

#### 3.2.1. Binding Mode of Quercetin 3-O-(6″-galloyl)-beta-D-galactopyranoside

**Figure 2:**
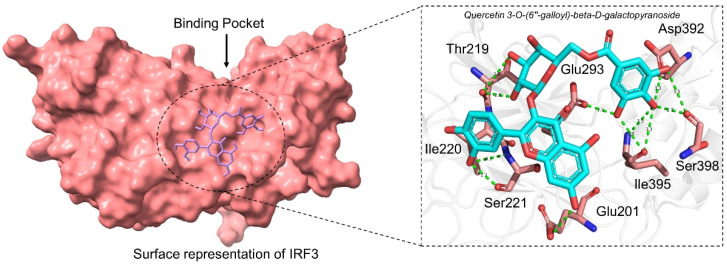
Binding mode of Quercetin 3-O-(6″-galloyl)-beta-D-galactopyranoside. The left panel show the surface representation of IRF3 and the binding of ligand. The right panel shows the interaction pattern and number of hydrogen bonds formed by each residue.

Quercetin 3-O-(6″-galloyl)-beta-D-galactopyranoside (SANC00850) isolated from *Myrothamnus flabellifolia* Welw is a flavonoid used against respiratory diseases and urinary tract infections in African countries [48]. It has also been shown that this compound induces antiviral effects against herpes simplex virus type 1. Here, with the docking score −6.93 kcal/mol, it has demonstrated the best activity against the SARS-CoV-2 ORF8-IRF3 complex. As shown in Figure 2, the compound interacts with the key interface residues and establishes several hydrogen bonds. Among the hydrogen bonding residues Glu201, Thr219, Ile220, Ser221, Glu293, ile395, Asp392, and Ser398 are involved. In this complex the [O2] of Glu201 interacts with the [O-] acting as acceptor here of the ligand. The [O2] of Thr219 amino acids contacted with [O2] at position 508 of the ligand atom. Furthermore the interactions formed by these atoms of amino acids and SANC00580 were reported to be responsible for the strongest binding 3722 [O3]...521 [O2], 551 [O2]...3724 [O2], 3724 [O2]...554 [O2], 3712 [O2]...3157 [O.co2], 3200 [O2]...3714 [O2], and 3714 [O2]...3247 [O2], respectively. For instance, these residues are previously reported to stabilize the IRF3-ORF8 complex, thus showing the inhibitory potential of this compound [6]. The binding mode of SANC00850 with IRF3 is shown in Figure 2.

#### 3.2.2. Binding Mode of Rutin

**Figure 3:**
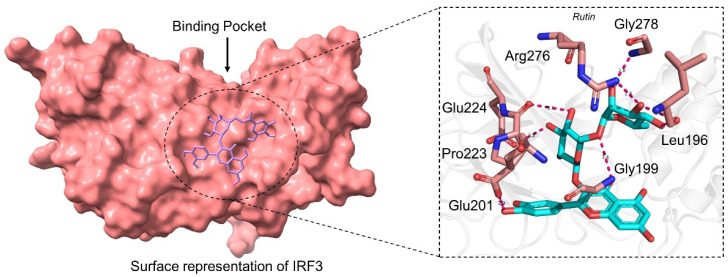
Binding mode of Rutin (SANC01085). The left panel show the surface representation of IRF3 and the binding of ligand. The right panel shows the interaction pattern and number of hydrogen bonds formed by each residue.

Rutin (SANC01085) or Rutoside or Quercetin 3-O-rutinoside is a flavonoid isolated from various plants that exhibit hepatoprotective, anti-inflammatory, and anti-microbial properties. It was originally isolated from Aspalathus linearis, a south African tea widely used against cytochrome P450 [49]. This compound possesses a similar scaffold as Quercetin 3-O-(6″-galloyl)-beta-D-galactopyranoside. The docking score for Rutin was −6.37 kcal/mol with seven hydrogen bonds established by 196Leu 3726 [O3]...137 [O2], 196Leu 3724 [O3]...137 [O2], 199Gly 183 [Nam]...3698 [O3], 201Glu 3712 [O2]...214 [O-], 223Pro 3720 [O3]...576 [O2], 224Glu 3718 [O3]...594 [O.co2], 224Glu 3716 [O3]...594 [O.co2], 276Arg 3722 [O3]...1372 [Ng+], 276Arg 1371 [Ng+]...3724 [O3], 276Arg 1372 [Ng+]...3724 [O3], 278Gly 1396 [Nam]...3722 [O], respectively. Among these, most of the interacting residues are conserved between Rutin and Quercetin 3-O-(6″-galloyl)-beta-D-galactopyranoside, thus justify the inhibition potential of these compounds. The binding mode of SANC01085 with IRF3 is shown in Figure 3.

#### 3.2.3. Binding Mode of Tribuloside

**Figure 4:**
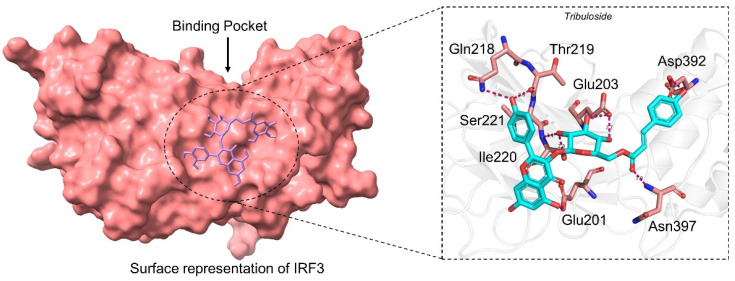
Binding mode of Rutin (SANC00867). The left panel shows the surface representation of IRF3 and the binding of the ligand. The right panel shows the interaction pattern and number of hydrogen bonds formed by each residue.

Tribuloside is also a flavonoid originally *Tribulus terrestris L* and characterized as an anti-mycobacterial compound. It is also known as kaempferol-3-β-d-(6″-p-coumaroyl) glucoside which is a derivate of Kaempferol [50]. Kaempferol has been reported to potentially inhibit the SARS-CoV-2 main protease (3CLpro), thus hinder the proteolysis of non-structural proteins [13,40]. Tribuloside with the docking score −6.27 kcal/mol formed nine hydrogen bonds with the key residues including Glu201, Glu203, Gln218, Thr219, Ile220, Ser221, Asp392, and Asn397. Deep interaction analysis revealed 201GLU 3708 [O2]...214 [O-], 203GLU 3716 [O3]...252 [O.co2], 203GLU 3718 [O3]...252 [O.co2], 218GLN 508 [Nam]...3712 [O2], 219THR 3712 [O2]...521 [O2], 221SER 551 [Nam]...3714 [O3], 221SER 3714 [O3]...556 [O3], 392ASP 3720 [O2]...3158 [O-] and 397ASN 3230 [Nam]...3696 [O2] formed interactions, respectively. This shows the inhibitory potential of these compounds against the IRF3-ORF8 complex. The binding mode of SANC01085 with IRF3 is shown in Figure 4.

### 3.3. Structural Dynamic Features of the Best Hits Complexes

#### 3.3.1. Structural Stability Assessment

**Figure 5:**
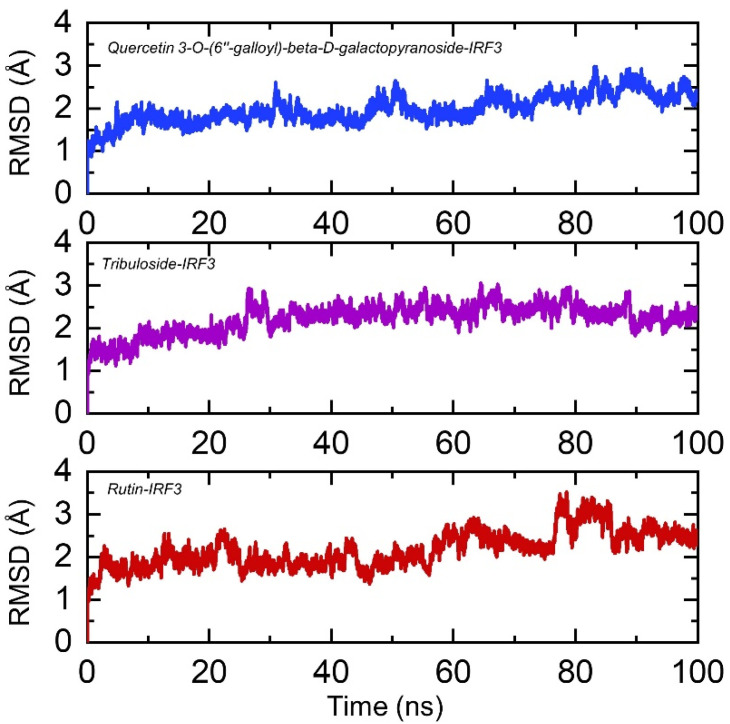
Dynamic stability of IRF3-Quercetin 3-O-(6″-galloyl)-beta-D-galactopyranoside, Tribuloside and Rutin complexes calculated as RMSD. The *x*-axis shows time in nanoseconds while *y*-axis show RMSD in Å.

Dynamic stability of a ligand-protein complex is very important to demonstrate the inhibitory potential of a drug. Thus herein, we used root mean square deviation (RMSD) as a function of time to estimate the stability of each protein-ligand complex over 100 ns simulation time period. As given in Figure 5, it can be seen that all the complexes remained stable during the simulation with little deviations at different time intervals were experienced by the complexes. In the case of Quercetin 3-O-(6″-galloyl)-beta-D-galactopyranoside-IRF3 complex, the system reached stability at 1.5 Å and at 5 ns. The structure did not face any convergence during the first 35 ns, however, a little deviation between 35–38 ns was showed then the structure remained stable. At different time intervals, particularly between 50–70 ns, faced a little perturbation, but overall, the complex remained stable. This shows the stable binding of Quercetin 3-O-(6″-galloyl)-beta-D-galactopyranoside to the IRF3 interface. On the other side, the Tribuloside-IRF3 complex remained comparatively more stable than the Quercetin 3-O-(6″-galloyl)-beta-D-galactopyranoside-IRF3 complex. Although the RMSD increased gradually but the average RMSD remained lower than 2.0 Å. A small deviation between 30–35 ns was experienced by Tribuloside-IRF3 complex but the complex dynamically remained stable. Moreover, the Rutin-IRF3 complex reported structural perturbation at different time intervals, particularly between 80–88 ns. Despite the structural deviation during the simulation, the average RMSD for this complex also remained lower (~2.2 Å). Consequently, this shows that all the compounds are stably bound to the target interface residues and exhibit the potential inhibitory properties. The RMSDs of all the complexes are shown in Figure 5.

#### 3.3.2. Structural Compactness Calculation

**Figure 6:**
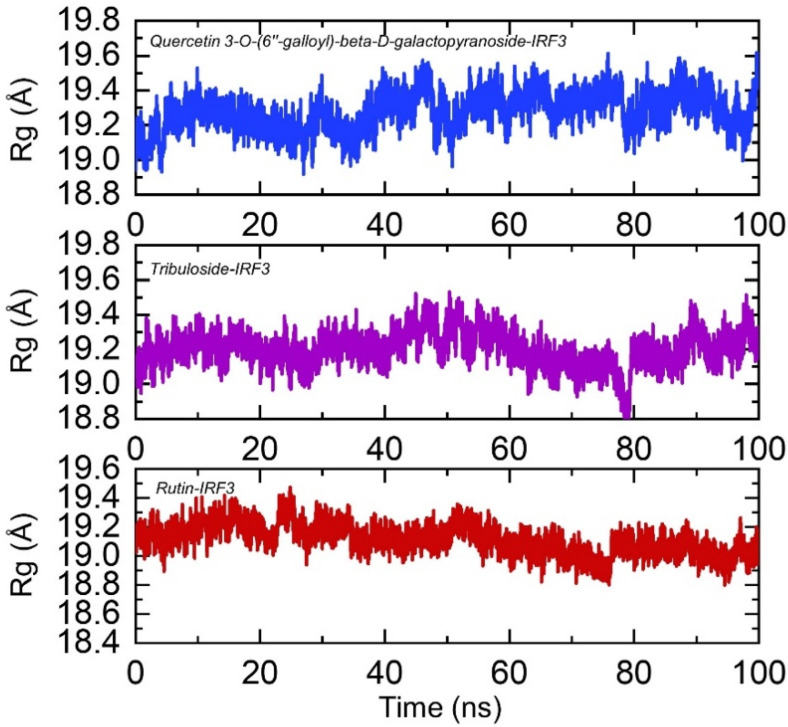
Structural compactness of IRF3-Quercetin 3-O-(6″-galloyl)-beta-D-galactopyranoside, Tribuloside and Rutin complexes calculated as Rg. The *x*-axis shows time in nanoseconds while *y*-axis show Rg in Å.

Understanding the structural compactness in a dynamic environment using simulation helps in understanding the packing of protein-ligand complexes and demonstrates the binding and unbinding events that happened during the simulation. Herein, the radius of gyration (Rg) was estimated for each complex as a function of time. As given in Figure 6, the structural compactness results are in strong uniformity with the RMSD results. In the case of IRF3-Quercetin 3-O-(6″-galloyl)-beta-D-galactopyranoside complex, the average Rg was reported to be 19.2 Å. Initially, the Rg increased until 10 ns but then decreased back and remained consistent until 30 ns. A significant drift was experienced between 40–50 ns, but then the structure remained compact. On the other hand, IRF3-Tribuloside demonstrated uniform results such as RMSD by remaining more compact throughout the simulation. The average Rg value was reported to be 19.0 Å for IRF3-Tribuloside complex. Furthermore, IRF3-Rutin complex displayed a higher Rg during the first 30 ns but then gradually decreased, and the average Rg was reported to be 19.1 Å. This shows that despite the binding and unbinding events, the top hits molecules stably bound the target protein steered by different bonds and biochemical events. The Rg(s) of all the complexes are shown in Figure 6.

#### 3.3.3. Residual Flexibility Estimation

**Figure 7:**
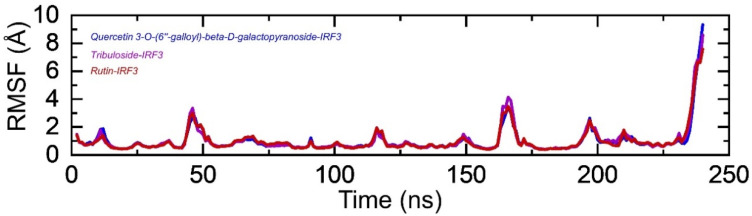
Residual flexibility of IRF3-Quercetin 3-O-(6″-galloyl)-beta-D-galactopyranoside, Tribuloside and Rutin complexes calculated as RMSF. The *x*-axis shows total residues while *y*-axis show RMSF in Å.

We computed the residual flexibility of all the complexes to provide an understanding of the strength contributed by each interacting residue in intermolecular association, the molecular recognition, and the probable effect on the global function of the biological molecules. Herein we calculated the residual flexibility as a root mean square fluctuation (RMSF). The results revealed that all the complexes exhibit minimal fluctuation stabilized by the binding of these molecules except at different regions where uniformly higher fluctuation was observed. These regions were witnessed as loops, and particularly, the terminal loop demonstrated higher fluctuation. Among the higher fluctuating regions, 40–50, 162–173, 190–200, and 235–240 demonstrated higher fluctuation. The RMSFs of all the complexes are shown in Figure 7.

#### 3.3.4. Hydrogen Bonding Analysis

**Figure 8:**
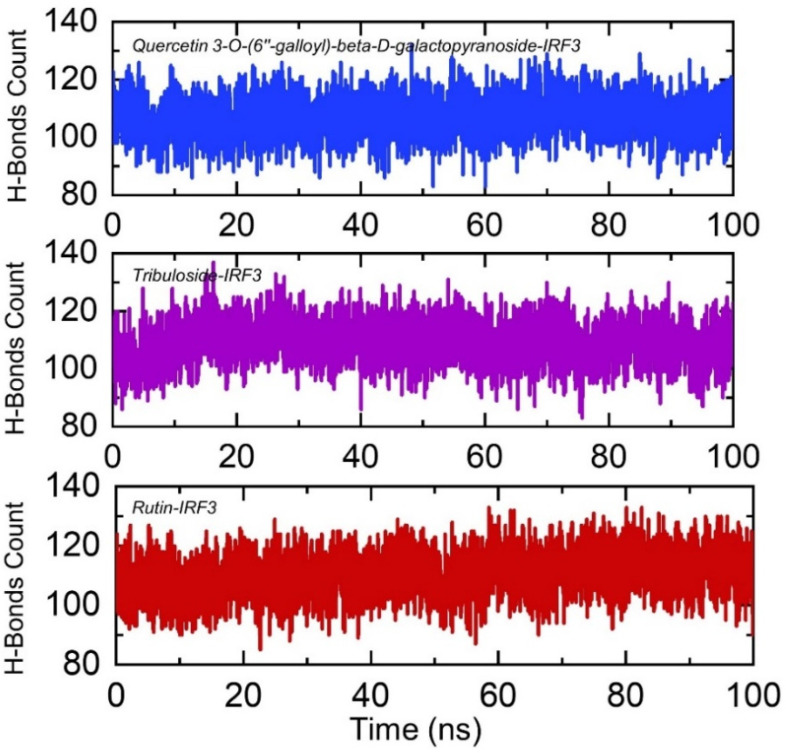
Hydrogen bonds estimation in IRF3-Quercetin 3-O-(6″-galloyl)-beta-D-galactopyranoside, Tribuloside and Rutin complexes. The *x*-axis shows time in nanoseconds while *y*-axis show hydrogen bonds count.

Protein-drugs association is primarily directed by a number of factors, among which hydrogen bonds and hydrophobic interactions are vital components. Hydrogen bonds in the protein interfaces are always engaged by water molecules which strive with the hydrogen bonding between the molecules [51]. The processes behind protein-protein/drugs coupling, as well as the implications to which hydrogen bonds play a role in this association, are very important [52]. Therefore, to comprehend the bonding pattern between the IRF3, Quercetin 3-O-(6″-galloyl)-beta-D-galactopyranoside, Rutin and Tribuloside, post-simulation hydrogen bonding analysis of each complex was performed to inform the binding specificity and biochemical events steered by hydrogen bonding. Herein, we calculated the total number of hydrogen bonds in each complex as a function of time. In the case of Quercetin 3-O-(6″-galloyl)-beta-D-galactopyranoside-IRF3 complex the average number of hydrogen bonds was reported to be 115, the average number of hydrogen bonds in Tribuloside-IRF3 complex were 113, while in Rutin-IRF3 complex, the average hydrogen bonds were also reported to be 115. This shows that hydrogen bonds formation and breakage events took place during the simulation. The total number of hydrogen bonds in all the complexes is shown in Figure 8.

### 3.4. Binding Free Energy Calculation

Estimation of binding free energy at the residue level of protein-ligand(s) complexes is the most frequently used approach in discovering small molecule inhibitors using in silico methods. This approach cross-validates the docking scores and re-evaluates the small molecule’s inhibitory potential interacting with a targeted protein. They are more comparatively more reliable than conventional docking scoring functions and computationally inexpensive compared to other methods such as Alchemical free energy approaches [53]. To estimate the binding free energies of all the complexes including vdW, electrostatic, SA, Gb and the total binding energy each trajectory was subjected to free energy calculation using MM/GBSA approach. The binding free energy calculation results revealed that for each complex the vdW was reported to be −34.86 kcal/mol (SANC00850-IRF3), −25.20 kcal/mol (SANC01085-IRF3) and −36.84 kcal/mol (SANC00867-IRF3). The electrostatic energy for each complex was reported to be −27.89 kcal/mol, −29.92 kcal/mol and −35.38 kcal/mol, respectively. The total binding energy given conclusive insights revealed that the total binding energy for SANC00850-IRF3 complex was −41.41 kcal/mol. For SANC01085-IRF3 the total binding energy was −40.33 kcal/mol while −40.62 kcal/mol was reported for SANC00867-IRF3. The fixation of the tails and backbone of the SANC00850 in the binding pocket gives it the advantage to bind robustly and inhibits the IRF3. It can be seen that 11 hydrogen bonds were more in number in Quercetin 3-O-(6″-galloyl)-beta-D-galactopyranoside-IRF3 complex because of the binding of whole scaffold in the active site. Moreover, the whole ligand is bound by the hydrogen bonds such as the two tails and the backbone. On the hand, the two ligands in the active sites are free either at one end or at the central backbone. Thus demonstrating the differences in the binding. The hydrogen bonding analysis also reports that hydrogen bonding reprogramming took place during the simulation and the number of average bonds validate the docking predictions. This data shows that these compounds interact with the interface residues of IRF3 more robustly, thus hinder the binding of partner protein ORF8 and rescue the host immune system. The MM/GBSA results for all the complexes are given in Table 2.

### 3.5. Bioactivity Prediction

Molinspiration predicted the bioactivity of the top hits. For Quercetin 3-O-(6″-galloyl)-beta-D-galactopyranoside the predicted bioactivity score was −0.05, for Tribuloside the predicted bioactivity score was 0.05 and for Rutin, the predicted bioactivity was 0.12. This shows the strongest inhibitory potential of these compounds because the server predicts scores for different compounds where the score ranges from −0.5 to 0.5 demonstrate excellent inhibitory activity. This approach has been previously used to estimate the bioactivity of potential compounds against SARS-CoV-2 [20].

### 3.6. Dissociation Constant Estimation (K_D_)

K_D_ is an important parameter to compute the binding strength of a drug. Thus, employing the same approach, the dissociation constant of the top three hits was estimated. The results revealed that for the Quercetin 3-O-(6″-galloyl)-beta-D-galactopyranoside-IRF3 complex the ∆G was −8.3 kcal/mol, for Rutin-IRF3 complex, the ∆G was predicted to be −7.7 kcal/mol, while for Tribuloside-IRF3 complex, the ∆G was predicted to be −7.8 kcal/mol. It can be seen that 11 hydrogen bonds were more in number in Quercetin 3-O-(6″-galloyl)-beta-D-galactopyranoside-IRF3 complex because of the binding of whole scaffold in the active site. Moreover, the whole ligand is bound by the hydrogen bonds such as the two tails and the backbone. On the hand, the two ligands in the active sites are free either at one end or at the central backbone. Thus demonstrating the differences in the binding. This shows that these compounds potentially interact with the target protein residues and curtail their binding to the interface of the interacting partner.

## 4. Conclusions

In conclusion, the current study uses computational screening and simulation approaches to identify novel drugs against the ORF8-ORF3 complex. This complex has been reported to suppress the host immune system and increase the disease severity. Through molecular search, we reported three drugs Quercetin 3-O-(6″-galloyl)-beta-D-galactopyranoside, Tribuloside and Rutin, potentially interact with the interface residues and abrogate the complex. Structural-dynamics analysis validated the inhibitory potential of these compounds. The total binding energy given conclusive insights revealed that the total binding energy for SANC00850-IRF3 complex was −41.41 kcal/mol. For SANC01085-IRF3 the total binding energy was −40.33 kcal/mol while −40.62 kcal/mol was reported for SANC00867-IRF3. This data shows that these compounds interact with the interface residues of IRF3 more robustly, thus hinder the binding of partner protein ORF8 and rescue the host immune system. Thus, these compounds need further in vitro and in vivo validations to be used as therapeutics against SARS-CoV-2.

## Figures and Tables

**Table 1:** 2D structures, database ID, name and docking scores of the best hits.

2D Structure	SANCDB ID	Name	IFD Scores
** 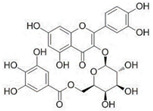 **	SANC00850	Quercetin 3-O-(6″-galloyl)-beta-D-galactopyranoside	−6.93
** 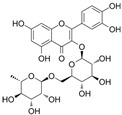 **	SANC01085	Rutin	−6.37
** 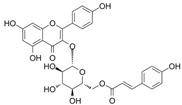 **	SANC00867	Tribuloside	−6.27

**Table 2:** MM/GBSA results for all the complexes including vdW, electrostatic and the total binding energy. All the energies are given in kcal/mol.

Complexes	vdW	Electrostatic	SA	GB	Total
SANC00850−IRF3	−34.86	−27.89	−5.30	26.65	−41.41
SANC01085−IRF3	−25.20	−29.92	−2.98	17.77	−40.33
SANC00867−IRF3	−36.84	−35.38	−3.10	34.70	−40.62

## Data Availability

The data presented in this study are available within the article.
